# Freeze-Driven Adsorption of Oligonucleotides with polyA-Anchors on Au@Pt Nanozyme

**DOI:** 10.3390/ijms251810108

**Published:** 2024-09-20

**Authors:** Nikita E. Lapshinov, Svetlana M. Pridvorova, Anatoly V. Zherdev, Boris B. Dzantiev, Irina V. Safenkova

**Affiliations:** A.N. Bach Institute of Biochemistry, Research Centre of Biotechnology of the Russian Academy of Sciences, 119071 Moscow, Russia; nikita_lapshinov@mail.ru (N.E.L.); sh-p_s@mail.ru (S.M.P.); zherdev@inbi.ras.ru (A.V.Z.); dzantiev@inbi.ras.ru (B.B.D.)

**Keywords:** freeze–thaw method, gold nanoparticles, lateral flow test, nanozymes, oligonucleotide conjugation, peroxidase-like activity, platinum nanoparticles, spherical nucleic acids

## Abstract

A promising and sought-after class of nanozymes for various applications is Pt-containing nanozymes, primarily Au@Pt, due to their easy preparation and remarkable catalytic properties. This study aimed to explore the freeze–thaw method for functionalizing Pt-containing nanozymes with oligonucleotides featuring a polyadenine anchor. Spherical gold nanoparticles ([Au]NPs) were synthesized and subsequently used as seeds to produce urchin-like Au@Pt nanoparticles ([Au@Pt]NPs) with an average diameter of 29.8 nm. The nanoparticles were conjugated with a series of non-thiolated DNA oligonucleotides, each consisting of three parts: a 5′-polyadenine anchor (A_n_, with n = 3, 5, 7, 10; triple-branched A_3_, or triple-branched A_5_), a random sequence of 23 nucleotides, and a linear polyT block consisting of seven deoxythymine residues. The resulting conjugates were characterized using transmission electron microscopy, spectroscopy, dynamic light scattering, and emission detection of the fluorescent label at the 3′-end of each oligonucleotide. The stability of the conjugates was found to depend on the type of oligonucleotide, with decreased stability in the row of [Au@Pt]NP conjugates with A_7_ > A_5_ > 3A_3_ > 3A_5_ > A_10_ > A_3_ anchors. These [Au@Pt]NP–oligonucleotide conjugates were further evaluated using lateral flow test strips to assess fluorescein-specific binding and peroxidase-like catalytic activity. Conjugates with A_3_, A_5_, A_7_, and 3A_3_ anchors showed the highest levels of signals of bound labels on test strips, exceeding conjugates in sensitivity by up to nine times. These findings hold significant potential for broad application in bioanalytical systems.

## 1. Introduction

Nanozymes, nanoparticles with catalytic activities similar to natural enzymes, have been actively studied in recent years and are considered as effective reagents for a wide range of applications due to their easy preparation, stability, resistance to inhibition, and wide range of conditions for catalysis [1,2]. One of the most popular areas of nanozyme application is their use in bioanalytical systems [3,4]. Ultrasensitive detection was demonstrated with the application of diverse groups of nanomaterials with catalytic properties such as noble metal nanomaterials and metal oxide nanomaterials [5,6,7], iron-based nanozymes [8], single-atom catalysts [9], and carbon-based nanomaterials [10]. Among them, bimetallic Pt-containing nanozymes are particularly in demand due to a larger surface area for biomolecule conjugation, distinctive optical properties (visually detectable black color) that provide an additional increase in signals, strong peroxidase-like activity, and straightforward synthesis protocols [11,12].

For their application in bioanalytical systems, nanozymes typically need to be conjugated with biomolecules that can specifically recognize a target or a secondary derivative component, which emerges from the target’s presence in a sample. Conjugates with oligonucleotides immobilized on the surface of nanoparticles allow combined molecular recognition due to programmable DNA/RNA and diversity possibilities for detection where optical, chemical, electrical, and catalytic properties of different nanoparticles are implemented [13,14,15]. Bimetallic Au@Pt nanoparticles ([Au@Pt]NPs) are considered as perspective nanozyme labels, and their conjugates with oligonucleotides have been successfully applied in recent developments of sensitive biosensors [16,17,18,19]. The advantages of [Au@Pt]NPs compared with traditional mono-compound nanoparticles including Au nanoparticles ([Au]NPs) are caused by such factors as a large surface area that facilitates contacts with substrates for catalytic redox reactions [20] and the generation of hot electron–hole pairs, significantly boosting peroxidase-like activity [21]. The increased catalytic activity of the [Au@Pt]NPs provides a background for lower detection limits of target analytes. The increasing demands for nanoparticle–oligonucleotide conjugates are further driven by the development of novel amplification methods, such as CRISPR/Cas12a, amplification approaches based on self-assembling DNA structures according to toehold-mediated strand displacement, hybridization chain reactions, catalytic hairpin assembly, entropy-driven catalysis, and others, where the oligonucleotide on the nanozyme surface is included in systems of DNA recognition or amplification [22,23].

Several works have reported simultaneous synthesis of nanozymes and nanozyme–DNA oligonucleotide conjugates by adding both the components required for nanozyme synthesis (noble metal salts and corresponding reducing agents) and the oligonucleotide at once [24,25]. However, protocols involving sequential synthesis—first the nanozyme, then the conjugate—allow for more precise control over the synthesis process and the homogeneity of the final product. Considerable experience has been accumulated in the syntheses of conjugates with oligonucleotides on [Au]NPs. The attachment to the surface is mainly due to thiol groups or polyadenine nucleotides (polyA), which have a high affinity for the gold surface [26,27,28,29]. However, Pt-containing nanoparticles differ from [Au]NPs in both the surface structure and chemical composition. Therefore, a targeted search for the best solutions for the immobilization of oligonucleotides on Pt-containing surfaces is needed. Even for AuNPs and oligonucleotides, immobilization presents a challenge due to the strong negative charge of both interacting compounds [30]. It is essential to find conditions that facilitate the proximity of the oligonucleotide and the nanoparticle while avoiding aggregation, which is likely under such syntheses [31]. Four main approaches exist for [Au]NP and oligonucleotide conjugation: (1) salt-aging and low-pH immobilization [32,33,34,35,36]; (2) the butanol dehydration method [37]; (3) microwave-assisted immobilization [38]; and (4) freeze–thaw adsorption [31,39].

Freeze–thaw adsorption is one-step method that allows for reproducible conjugate synthesis under well-standardized temperature conditions regardless of the volume of the synthesized conjugate (microwave-assisted immobilization). It requires only 1 h at −20 °C and does not require additional reagents such as salt or butanol (in comparison with salt-aging and low-pH immobilization and butanol dehydration methods), allowing the creation of stable conjugates with a high density of oligonucleotides on the [Au]NP surface [31,40,41,42,43]. This method was first successfully employed for the synthesis of conjugates between [Au]NPs and thiolated oligonucleotides [39]. The immobilization processes in this method are considered according to Liu B. and Liu J. [39] as a result of the formation of “micropockets” between ice crystals where AuNPs, DNA, and salts (Na^+^, citrate ions) are concentrated and conjugated with higher effectiveness. In a subsequent study, the developers of the freeze–thaw method, Liu et al., demonstrated that a key feature of the method is the stretching and alignment of oligonucleotides that occur at sub-zero temperatures [44]. For [Au]NP–oligonucleotide conjugates, it was shown that the method is effective even without sulfur-containing groups and works well for single-stranded DNA when a polyA block (at the 5′-end, for nanoparticle attachment) and a polyT block (3′-end, ensuring conjugate stability) are simultaneously present [31,38]. The salt-aging, low-pH, and freezing methods were used to synthesize conjugates of Pt-containing nanozymes with oligonucleotides only in the presence of a thiol group [16,17,19,45,46]. However, conjugates of nanozymes and oligonucleotides without thiol groups are also in great demand, since they are significantly cheaper to oligonucleotide-synthesize and do not require additional treatment to activate disulfide bonds. To our knowledge, no study has been conducted on the freeze–thaw immobilization of poly-A-anchoring oligonucleotides on the Pt surface of nanozymes that would allow the selection and recommendation of the best conditions for the conjugate synthesis. Therefore, comprehensive studies are needed to evaluate the effectiveness of the freeze–thaw method concerning Pt-containing nanozymes and oligonucleotides with polyA anchors.

Herein, the freeze–thaw method was applied to functionalize Pt-containing nanozymes with oligonucleotides to preserve optical and catalytic properties of the nanozymes and the functional properties of the oligonucleotides. This study was conducted using one of the most common Pt-containing nanozymes—urchin-like bimetallic [Au@Pt]NPs, produced by reducing Pt^4+^ ions on the surface of [Au]NPs [20]. This nanozyme is characterized by a large surface area due to its rough, spiky surface and high peroxidase-like catalytic activity [40]. [Au@Pt]NPs and their derivatives have found wide applications, for example, in in vitro reactions, enabling quantitative catalytic oxidation reactions based on typical peroxidase substrates. To obtain the conjugate, we selected six oligonucleotides differing in their polyA block—5′-poly-adenine anchors (A_n_, with n = 3, 5, 7, 10; triple-branched A_3_ and triple-branched A_5_). The [Au@Pt]NP–oligonucleotide conjugates were obtained using the freeze–thaw method, which retained catalytic activity and interaction capability due to the functional label (fluorescein) at the terminal opposite to the nanoparticle attachment. The effectiveness of the conjugation was found to depend on the length and structure of the 5′-poly-adenine anchor. To our knowledge, this study represents the first report of [Au@Pt]NP–oligonucleotide conjugates obtained by the freeze–thaw method based on oligonucleotides with polyA and polyT blocks. The novelty of this study consists in demonstrating the advantages of short polyA anchors for Pt-containing surfaces, which differ from the known optimal length of polyA anchors interacting with Au-containing surfaces.

## 2. Results and Discussion

### 2.1. Design of Experiments

**Figure 1:**
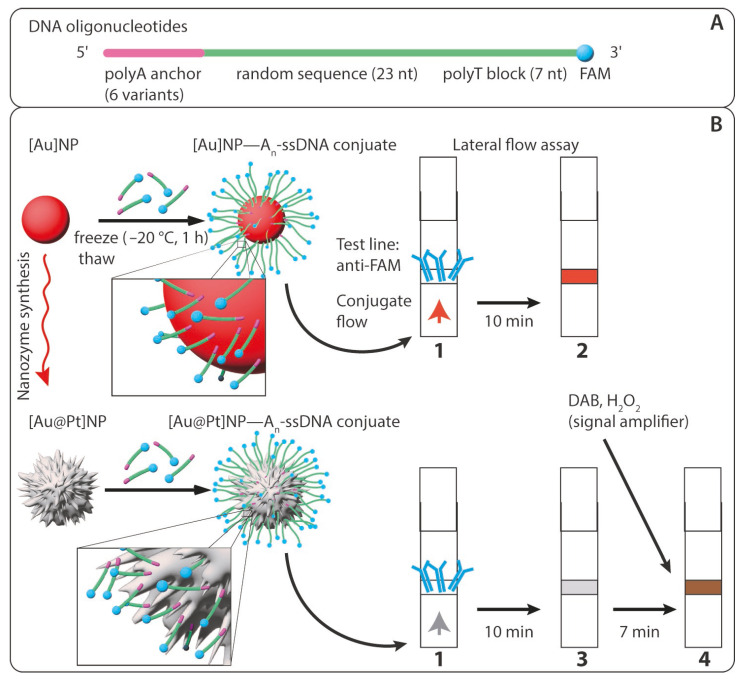
Schemes of the experiments. (**A**) Oligonucleotide (A_n_-ssDNA) for immobilization consisting of 5′-polyadenine anchor (A_n_, with n = 3, 5, 7, 10; triple-branched A_3_ and triple-branched A_5_), random sequence, polythymine block, fluorescein; (**B**) syntheses of conjugates and testing of their binding capacity using lateral flow assay (1—lateral flow test strip with anti-FAM immobilized in the binding zone before assay; 2—test strip with red line due to the binding of [Au]NP conjugate in the binding zone; 3—test strip with gray line due to the binding of [Au@Pt]NP conjugate in the binding zone; 4—test strip with brown line due to the peroxidase-like catalytic properties of [Au@Pt]NP conjugate in the binding zone). FAM—fluorescein; anti-FAM—monoclonal antibodies specific to fluorescein; DAB—3,3′-diaminobenzidine.

The primary objective of this study was to develop a conjugate of [Au@Pt]NP–oligonucleotides that retains the optical and catalytic properties of the nanozyme while enabling interaction through oligonucleotides. As the methodological basis for synthesizing this conjugate, we chose the freeze–thaw method, which has previously demonstrated its efficacy [31,40,41,42,43] for conjugating [Au]NPs with non-thiolated DNA oligonucleotides containing terminal polyA blocks for attachment. This method has not been previously applied to Au@Pt nanozymes and non-thiolated DNA oligonucleotides. For a comprehensive comparative study, we selected [Au]NPs of small diameter (10 nm) and [Au@Pt]NP nanozymes, synthesized by reducing Pt^4+^ on the surface of these gold nanoparticles. For immobilization, we used a series of oligonucleotides, each consisting of three parts (as depicted in Figure 1A), described below in the 5′-3′ direction:

(1) A linear polyA block consisting of 3, 5, 7, or 10 deoxyadenine residues (A_n_), triple-branched A3 (3A_3_), or triple-branched A5 (3A_5_): The triple-branched structures were obtained using trebler phosphoramidite during oligonucleotide synthesis. The polyA block is necessary for attaching the oligonucleotide to the nanoparticle surface, as deoxyadenine has the highest affinity for gold [31,38]. A_7_ anchors are sufficient for robust attachment of oligonucleotides to [Au]NP surfaces when an additional polyT block (at least T_5_) is present at the opposite end [31]. In our study, the triple-branched A block was used as an anchor for the first time, and we hypothesized that it would provide better binding of the oligonucleotide to the nanoparticle surface.
(2) A random sequence of 23 nucleotides: This length refers to the interval that covers most of the sequences used in molecular genetic analysis for unique hybridization interactions or selective enzymatic hydrolysis.
(3) A polyT block consisting of seven linear deoxythymine residues (T_7_): The polyT block does not adsorb onto the nanoparticle surface due to its minimal affinity in the order of A > C > G > T [47,48]. However, several studies have shown that the presence of a polyT block as a tail at the opposite end of the polyA block, or even as a spacer immediately following the polyA block, significantly improves the adsorption of the oligonucleotide and stabilizes the conjugate [31,38].

Each oligonucleotide was labeled with a fluorescein (FAM) at the 3′-end. The FAM label served two purposes: to monitor the efficiency of oligonucleotide adsorption on the nanoparticle surface and to verify the conjugate’s binding capability through the oligonucleotide. To assess the binding capability of the conjugate, as well as the optical and catalytic properties of the nanozyme, we utilized a lateral flow assay (LFA). In the binding zone, antibodies specific to fluorescein were immobilized, allowing the conjugate to bind via FAM and stain the zone red due to [Au]NPs or black due to the nanozyme. The peroxidase-like catalytic properties were evaluated using the 3,3′-diaminobenzidine (DAB) substrate (schemes of the experiments are presented in Figure 1B).

### 2.2. Synthesis and Characterization of Nanoparticles

**Figure 2:**
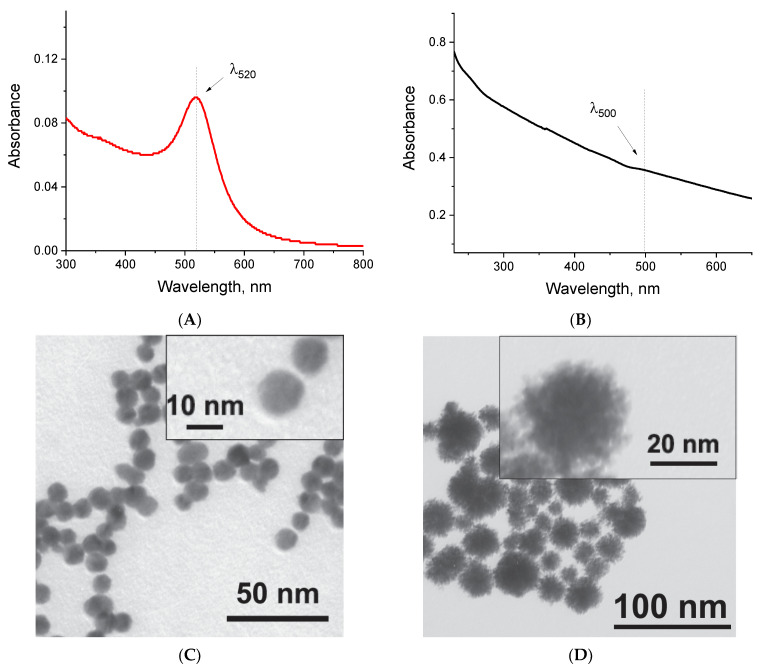

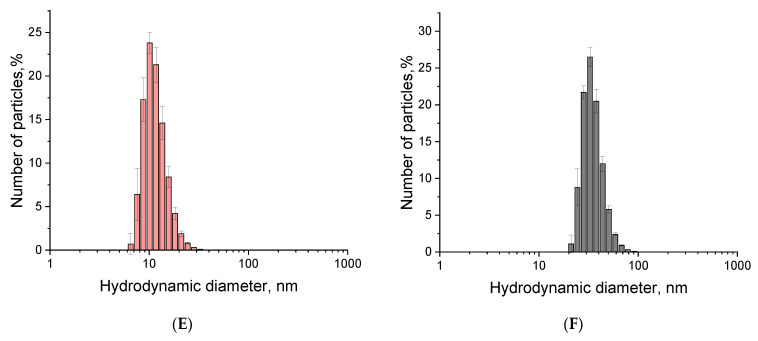
Characterization of the synthesized nanoparticles. Absorption spectra of [Au]NPs (**A**) and [Au@Pt]NPs (**B**); TEM micrographs of [Au]NPs (**C**) and [Au@Pt]NPs (**D**); hydrodynamic diameter distributions obtained by DLS for [Au]NPs (**E**) and [Au@Pt]NPs (**F**).

The first step involved the synthesis of [Au]NPs using the citrate reduction of Au^3+^ cations from tetrachloroauric acid. Figure 2A shows the absorption spectrum in the UV/visible region (300–800 nm) of the obtained [Au]NP solution, which has a bright red color. The absorption maximum corresponds to a wavelength of 518–520 nm, attributed to the surface plasmon resonance. The wavelength of 520 nm was selected for the quantitative assessment of nanoparticles in the conjugate solution. The size and homogeneity of the particles were evaluated using transmission electron microscopy (TEM) and dynamic light scattering (DLS). TEM revealed that the [Au]NPs have a spherical shape with an average diameter of 10.6 ± 3.6 nm (the micrograph of the nanoparticles is presented in Figure 2C, and the diameter distribution histogram is shown in Figure S1A). DLS confirmed the homogeneity of the nanoparticles in the solution, showing a hydrodynamic diameter of 11.8 ± 3.5 nm with a polydispersity index (PdI%) of 0.478 (Figure 2E).

Based on the synthesized [Au]NPs, we conducted the synthesis of [Au@Pt]NPs by reducing Pt^4+^ cations of sodium hexachloroplatinate with sodium ascorbate, using proportions of [Au]NPs/Pt^4+^/ascorbic acid that maximized the peroxidase-mimicking activity of the nanozyme according to Panferov et al. [49]. Simultaneous determination of Pt and Au contents in the synthesized [Au@Pt]NPs, as well as Au determination in the synthesized [Au]NPs, was successfully performed using X-Ray Fluorescence (XRF) spectrometry. The resulting [Au@Pt]NP solution was black and did not exhibit a characteristic absorption peak in the spectrum (Figure 2B). For the quantitative assessment of nanoparticles in the conjugate solution, we selected absorption at a wavelength of 500 nm. TEM characterization of [Au@Pt]NPs confirmed that the particles are spherical with a spiky surface (Figure 2D). The spiky surface increases the catalytic surface area of the nanozyme due to the larger number of catalytic centers, as was shown earlier when comparing a number of bimetallic nanozymes that differ in surface structure [49]. However, the significant roughness of the nanozyme surface can affect the binding of oligonucleotides due to several adenines. The diameter of [Au@Pt]NPs was 29.8 ± 5.6 nm (the diameter distribution histogram is presented in Figure S1B). DLS confirmed the homogeneity of the nanoparticles in solution, revealing a single peak with a hydrodynamic diameter of 35.4 ± 9.1 nm and a PdI% of 0.101 (Figure 2F).

For [Au@Pt]NPs, we evaluated peroxidase-like properties in solution using substrates such as 3,3′,5,5′-tetramethylbenzidine (TMB) and hydrogen peroxide. The nanozyme catalyzed the oxidation of TMB turned it into a blue-colored product. The characteristic reaction was observed and detected after 15 min in a dilution range of nanozyme from 450 to 36,500 (the results are presented in Figure S2).

### 2.3. Synthesis and Characterization of Nanoparticle–Oligonucleotide Conjugates

For both types of nanoparticles, we synthesized their conjugates with oligonucleotides differing in the length and structure of the polyA block (the sequences are presented in Table 1). The conjugation was performed using the freeze–thaw method with incubation at −20 °C for 1 h. The resulting conjugates ([Au]NP–A_n_-ssDNA, [Au@Pt]NP–A_n_-ssDNA) were separated from free oligonucleotides by centrifugation, and the immobilization efficiency was assessed by measuring the FAM fluorescence in the supernatants. Fluorescence spectra showed that after the third centrifugation, no free oligonucleotide remained in the conjugate preparation (fluorescence spectra of supernatants are presented in Figure S3). The resulting conjugates, as well as their components (nanoparticles, oligonucleotides), were characterized by Fourier transform infrared (FTIR) spectroscopy (Figure S4). The spectrum of the conjugate showed bands in the regions of 1680–1580 cm^−1^, 1130–830 cm^−1^, and 530–450 cm^−1^ associated with the absorption of nucleotides immobilized on a gold/platinum surface [47,50]. Thus, FTIR spectroscopy data confirm the successful preparation of the [Au@Pt]NP–oligonucleotide conjugates.

**Figure 3:**
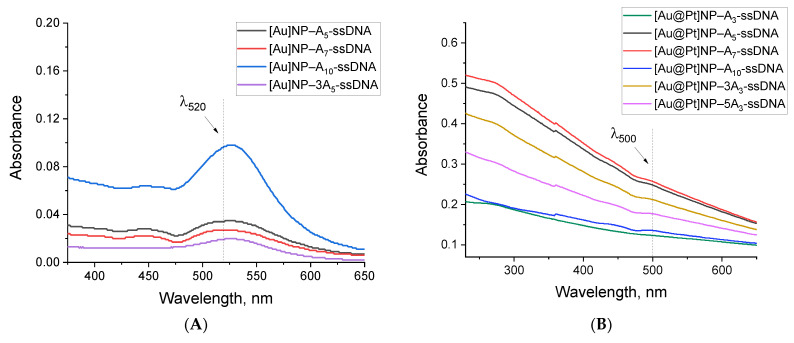

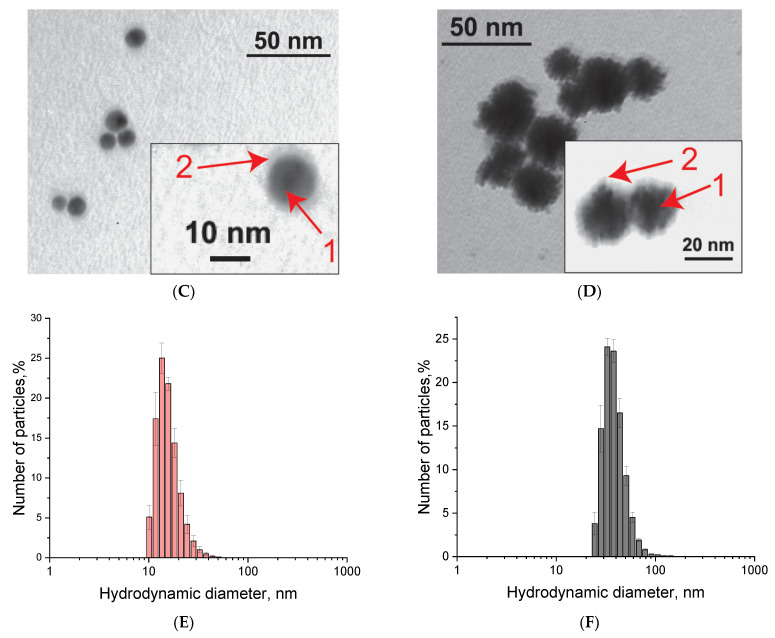
Characterization of synthesized conjugates. Absorption spectra of [Au]NP–A_n_-ssDNA (**A**) and [Au@Pt]NP–A_n_-ssDNA (**B**); TEM micrographs of [Au]NP–A_10_-ssDNA (**C**) and [Au@Pt]NP–A_7_-ssDNA (**D**); hydrodynamic diameter distributions obtained by DLS for [Au]NP–A_10_-ssDNA (**E**) and [Au@Pt]NP–A_7_-ssDNA (**F**). Arrow 1 shows the nanoparticle; arrow 2 shows the immobilized oligonucleotide.

Spectrophotometric characterization of the conjugates revealed that absorption at 520 nm for [Au]NPs and 500 nm for [Au@Pt]NPs (the selected wavelengths for nanoparticle assessment in conjugates) depended on the polyA block (Figure 3). The lower the absorption, the greater the losses due to aggregation during synthesis. For [Au]NPs, maximum absorption was observed for conjugates with A_10_, while for [Au@Pt]NPs, A_7_ > A_5_ > 3A_3_ > 3A_5_. Thus, even a preliminary spectrophotometric comparison of the conjugates indicates that the structure of the polyA block, which ensures greater conjugate stability, depends on the type of nanoparticles.

TEM characterization was performed for all conjugates (micrographs are shown in Figure 3C,D and Figures S5 and S6). For some conjugates ([Au]NP–A_10_-ssDNA, [Au@Pt]NP–A_7_-ssDNA), a clear rim corresponding to an oligonucleotide shell around nanoparticles was observed. Thus, the shell was most pronounced in the conjugates that exhibited greater stability. DLS characterization of the conjugates showed an increase in average hydrodynamic size relative to nanoparticles. The degree of this increase was varied depending on the oligonucleotide used. For [Au]NP conjugates, a significant increase in hydrodynamic diameters to 21–23.9 nm was observed, corresponding to a change of 10–12.1 nm relative to nanoparticles. The largest size increase was observed for the [Au]NP–A_5_-ssDNA conjugate, likely due to a tendency toward aggregation.

For [Au@Pt]NP conjugates, the changes were less pronounced, with an increase of 2.1–10 nm relative to nanoparticles (hydrodynamic characteristics for all synthesized conjugates are presented in Table S1). The smaller change in hydrodynamic diameters for [Au@Pt]NP conjugates could be associated with the absence of aggregated nanoparticles, but the spiky surface of the conjugate may partially mask size changes caused by oligonucleotide immobilization. The distributions of the hydrodynamic diameters for the most stable conjugates, [Au]NP–A_10_-ssDNA and [Au@Pt]NP–A_7_-ssDNA, are presented in Figure 3E,F. The polydispersity index showed higher homogeneity for [Au@Pt]NP conjugates compared to [Au]NP conjugates (see Table S1). Thus, although conjugates were successfully obtained for all types of oligonucleotides, they differed in aggregation characteristics and stability, and the most stable and homogeneous conjugates were [Au]NP with A_10_-ssDNA and [Au@Pt]NP with A_7_-ssDNA. There are probably two reasons for the observed effect: firstly, platinum binds polyA tails more strongly, so even a smaller number of adenines is enough to form a strong connection, and secondly, the rough, spiky surface of the nanozyme hinders the binding of long polyA blocks.

The catalytic properties of the [Au@Pt]NP conjugates were confirmed in the same manner as for the nanoparticles using the reaction with TMB and hydrogen peroxide. A slight decrease in the catalytic activity of the conjugates compared to the nanoparticles was observed after 15 min of the reaction (Figure S2), with a dilution range of catalytically active conjugates from 450 to 36,500. The reduction in catalytic activity can be attributed to partial shielding of the surface by immobilized oligonucleotides.

### 2.4. Comparison of the Conjugates Using Lateral Flow Test Strips

The functional properties of the conjugates based on both types of nanoparticles were confirmed by the retention of the fluorescein-specific binding property of oligonucleotides (fluorescein group was located at the 3′-end of the oligonucleotides) after their immobilization on nanoparticles. The conjugates were tested using lateral flow strips where antibodies specific to fluorescein were immobilized in the binding zone. Such testing is the simplest and most effective way to compare the conjugates in terms of their binding capability. To achieve this, lateral flow test strips were prepared (see Section 3.6), and the conjugates were adjusted to the same optical density (A_520_ for [Au]NP and A_500_ for [Au@Pt]NP) to ensure the same number of conjugate particles. The conjugates were added to the buffer solution, into which the test strips were then dipped. Driven by capillary forces, the conjugates moved along the nitrocellulose membrane and reached the binding zone, forming fluorescein–antibody complexes.

For the [Au]NPs conjugates, the interaction described above occurred in the binding zone, resulting in its red staining. Densitometric analysis of the stained binding zones showed that the highest color intensity was observed for the [Au]NP–A_10_-ssDNA conjugates with oligonucleotides containing an A_10_ polyadenine block. The color intensity in the binding zone for conjugates with oligonucleotides with a shorter polyaA block was reduced significantly; it was from 16 to 28 times less intense (see Figures S7 and S8). These results confirm that for [Au]NP conjugates synthesized by the freeze–thaw method, increasing the length of the polyA anchor leads to more effective adsorption of the oligonucleotide on the nanoparticle surface. The use of a branched polyA anchor did not improve the efficiency of oligonucleotide immobilization.

**Figure 4:**
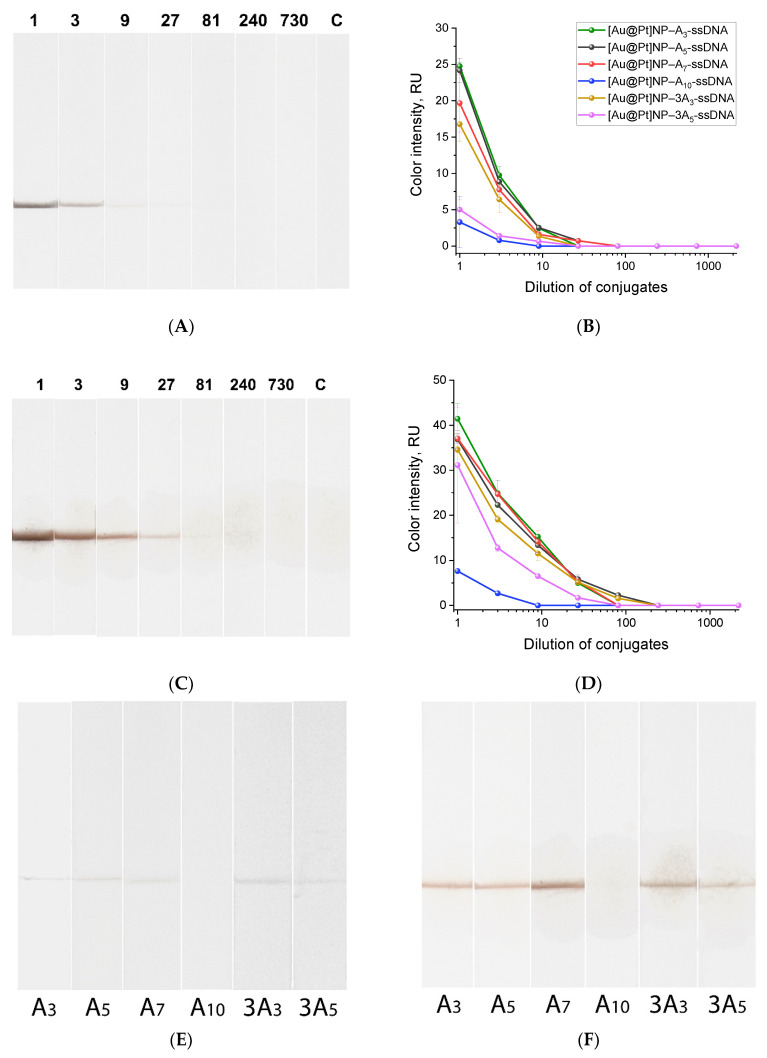
Test strips with binding zones stained by binding of [Au@Pt]NP–A_n_-ssDNA conjugates and the dependencies of binding zone coloration. (**A**) Test strips colored due to the colorimetric properties of nanozyme for [Au@Pt]NP–A_7_-ssDNA conjugates (the numbers indicate the dilution factor; C shows the control experiment without conjugate), and (**B**) dependencies of binding zone coloration on conjugate dilution (for each dilution, two replicates were made; the figure shows the mean values and standard deviations as error bars); (**C**) test strips with enhanced coloration due to catalytic properties of nanozyme for [Au@Pt]NP–A_7_-ssDNA (the numbers indicate the dilution factor; C shows the control experiment without the conjugate), and (**D**) dependencies of binding zone coloration on conjugate dilution (for each dilution, two replicates were made; the figure shows the mean values and standard deviations as error bars); (**E**) comparison of test strips for six conjugates at 9-fold dilution without catalytic enhancement; (**F**) comparison of test strips for six conjugates at 9-fold dilution after catalytic enhancement.

For the [Au@Pt]NP conjugates, black staining occurred in the binding zone, indicating the complexes’ formation. The highest color intensity accorded with the conjugates with oligonucleotides containing A_3_, A_5_, A_7_, and 3A_3_. Moreover, densitometric analysis of the test strips for conjugates at different dilutions showed that A_5_- and A_7_-ssDNA conjugates were detected in the binding zone even at dilutions of 81, which means a concentration 3–9 times lower relative to other conjugates (Figure 4A,B and Figure S9). A comparison of test strips for all six conjugates at 9-fold dilution is shown in Figure 4E. The following explanation can be made. The more oligonucleotides are immobilized on the surface of the nanozyme, the more effective the binding in the test zone will be due to the formation of a complex of fluorescein included in the oligonucleotide and antibodies specific to fluorescein immobilized in the binding zone of the test strip. The greater the amount of conjugate bound in the binding zone, the more intense the staining or catalytic reaction in the test zone. Thus, the color intensity of the test zone as a result of direct colorimetric or catalytically enhanced registration indicates the effectiveness of conjugate formation and the effectiveness of the polyA block as an anchor for immobilization.

Another important characteristic of the [Au@Pt]NP conjugate is the enhancement of coloration in the binding zone of the test strip due to nanozyme catalysis. To assess these characteristics, we used 3,3′-diaminobenzidine (DAB) and hydrogen peroxide—the substrates for horseradish peroxidase and peroxidase-like nanozymes that are commonly used in heterogeneous assays. During the redox reaction catalyzed by the nanozyme presenting in the binding zone, the zone turns brown due to the formation of the DAB derivative. Figure 4C shows test strips after analyzing different dilutions of the [Au@Pt]NP–A_7_-ssDNA conjugate (test strips for all conjugates are presented in Figure S9); the brown staining of the binding zones is due to the catalytic properties of the [Au@Pt]NPs. Figure 4D shows the relationship between dilution for all [Au@Pt]NP conjugates and the coloration of the binding zone. Thus, the peroxidase-like properties of the [Au@Pt]NP conjugates were confirmed under heterogeneous conditions on the nitrocellulose membrane of the test strip. A comparison of the dependencies in Figure 4B,D reveals that after adding the DAB and hydrogen peroxide mixture, the optical signal increased by approximately nine times due to brown coloration. A comparison of test strips after the signal enhancement for all six conjugates at 9-fold dilution is shown in Figure 4F. The conjugates with oligonucleotides containing A_3_, A_5_, A_7_, and 3A_3_-ssDNA showed high and comparable signals in binding zones, which were approximately nine times greater than those of the other two conjugates.

These results indicate that long (A_10_) or highly branched (3A_5_) polyA blocks are not optimal for functionalizing [Au@Pt]NPs. The most effective conjugates had shorter polyA blocks: A_3_, A_5_, A_7_, and 3A_3_-ssDNA. This differs from the optimal polyA block (A_10_) for functionalizing [Au]NPs. The likely reason for the reduced efficiency of [Au@Pt]NP conjugation with A_10_-ssDNA is the rough, spiky surface of the nanozyme, which hinders the binding of long polyA blocks. At the same time, binding to the Pt-containing surface with the polyA block is stronger, as even short A_3_- and A_5_-ssDNAs are able to provide a stable functional conjugate.

## 3. Materials and Methods

### 3.1. Materials

Oligonucleotides (sequences provided in Table 1) were custom-synthesized and purified by Lumiprobe (Moscow, Russia). The study used monoclonal mouse antibodies specific to fluorescein (anti-FAM) (Bialexa, Moscow, Russia); streptavidin (IMTEK, Moscow, Russia); sodium tetrachloroaurate and sodium hexachloroplatinate; sodium ascorbate; sodium citrate; sodium azide; bovine serum albumin (BSA) and 30% hydrogen peroxide (Sigma-Aldrich, St. Louis, MO, USA); Triton-X-100, Tween-20, and agarose (DIA-M, Moscow, Russia); glycerol (Reakhim, Moscow, Russia); a substrate mixture for peroxidase activity based on 3,3′,5,5′-tetramethylbenzidine (TMB) and hydrogen peroxide (Immunotech, Moscow, Russia); a substrate kit for peroxidase activity based on 3,3′-diaminobenzidine (DAB) and hydrogen peroxide (Servicebio, Wuhan, Hubei Province, China); and tris(hydroxymethyl)aminomethane (Loba Chemie, Mumbai, Maharashtra, India). All the salts and organic compounds used were of analytical-grade purity. A Simplicity Milli-Q^®^ system from Millipore (Burlington, MA, USA) was used to obtain ultrapure water for buffers and reagent solutions.

For the fabrication of lateral flow test strips, CN-95 nitrocellulose membranes (Sartorius Stedim Biotech, Göttingen, Germany), AP045 absorbent membrane, and L-P25 plastic backing (Advanced Microdevices, Ambala Cantt, Haryana, India) were used. Measurements of the catalytic activity of the nanozyme and its conjugates in solution were conducted using 96-well transparent polystyrene microplates (Nunc, Roskilde, Denmark).

### 3.2. Preparation and Characterization of Gold Nanoparticles

[Au]NPs were synthesized by the Frens method [51] with slight modifications. A filtered (0.22 μm) solution of 1% sodium citrate (3 mL) was added to 46.5 mL of deionized water. The mixture was continuously stirred and heated to boiling and boiled for 5 min, and then 0.5 mL of 1% HAuCl_4_ was added, followed by boiling for 30 min. The [Au]NP solution was then cooled and stored at 4 °C.

### 3.3. Preparation of Bimetallic Au@Pt Nanoparticles

[Au@Pt]NPs were synthesized according to the protocol described by Panferov et al. [49], with slight modifications. To 20 mL of the prepared gold nanoparticles (see Section 3.2), 4 mL of 10 mM sodium hexachloroplatinate was added. Then, 5.3 mL of distilled ultrapure water was added to the mixture, and it was heated to 80 °C for about 1 min in a water bath. Subsequently, 4 mL of 50 mM sodium ascorbate was introduced using a peristaltic pump at a rate of 400–500 μL/min (~8 min) and heated at 80 °C for 15–20 min. The nanozyme solution was then brought to room temperature and stored at 4 °C.

### 3.4. Nanoparticle Characterization

The sequential X-Ray Fluorescence Spectrometer ARL ADVANT’X IntelliPower 2500 (Thermo Scientific, Waltham, MA, USA) was used for total element analysis of synthesized [Au]NPs and [Au@Pt]NPs.

Spectra of the synthesized preparations were recorded on a Libra spectrophotometer (Biochrom, Cambourne, Cambridge, UK) in the wavelength range of 300 to 800 nm using cuvettes with optical path lengths of 0.1 cm. For characterization of nanoparticles by TEM, nanoparticle solutions (10 μL) were applied to 300-mesh copper grids (Pelco International; Redding, CA, USA) coated with a poly(vinyl formal) film, and adsorbed for 10 min, and then excess liquid was removed with filter paper and the samples were dried. Measurements were conducted on a JEM CX-100 transmission electron microscope (Jeol, Tokyo, Japan) at 80 kW and a magnification of 33,000–100,000. The resulting micrographs of nanoparticles were scanned, and the average diameter of nanoparticles was determined for no fewer than 100 nanoparticles in the sample. The hydrodynamic sizes of nanoparticles in the solution were measured by DLS using a Zetasizer Nano ZSP (Malvern Panalytical, Malvern, UK). All hydrodynamic size measurements were conducted in a disposable micro cuvette (ZEN0040; Malvern Panalytical, Malvern, UK) at 25 °C and a scattering angle of 173°. Hydrodynamic diameters and polydispersity indices (PdI%) were calculated automatically based on at least three repeats (each measurement consisted of 50 acquisitions) using Zetasizer Software v.8.00 (Malvern Panalytical, Malvern, UK).

To measure the catalytic activity of the nanozyme in solution, the synthesized [Au@Pt]NPs were titrated (from A_500_ = 0.07 (50-fold dilution of the nanozyme) in step 3, final volume 100 μL) in 50 mM phosphate buffer, pH 7.4, containing 100 mM NaCl and 0.05% Triton X-100 (PBST). Subsequently, 100 μL of TMB substrate mixture and an additional 20 μL of 30% hydrogen peroxide were added to the wells according to Panferov et al. [52]. The mixture was incubated at room temperature for 15 min, after which the reaction was stopped by adding 50 μL of 1 M H_2_SO_4_. The absorption in each well was recorded at a wavelength of 450 nm using an EnSpire multimode plate reader (PerkinElmer, Waltham, MA, USA).

### 3.5. Synthesis of Oligonucleotide–Nanoparticle Conjugates

Conjugate synthesis was conducted using the freeze–thaw method according to the protocol described by Wang et al. [31]. A mixture of 400 μL of synthesized nanoparticles and 20 μL of 100 μM oligonucleotides was placed in a freezer at −20 °C. After 1 h, the mixture was brought to room temperature and allowed to thaw. To separate the conjugate from unbound oligonucleotides, three rounds of centrifugation were performed at 16,000× *g* for [Au]NPs or 14,000× *g* for [Au@Pt]NPs at 4 °C for 20 min. The pellet, corresponding to the formed conjugate, was resuspended in 0.01 M phosphate buffer (0.1 M NaCl, pH 7.4) after the first and second centrifugations, and in 0.01 M phosphate buffer (0.3 M NaCl, pH 7.4) after the third centrifugation.

### 3.6. Fabrication of Lateral Flow Test Strips

The CN-95 nitrocellulose membrane was affixed to the L-P25 plastic backing, onto which anti-FAM at a concentration of 1 mg/mL in a 50 mM potassium phosphate buffer, with a pH of 7.4 with 100 mM of NaCl, 5% glycerol, and 0.03% NaN_3_, was applied on the nitrocellulose membrane at a deposition rate of 1.5 μL/mm using an IsoFlow dispenser (Imagene Technology, St. Lebanon, NH, USA). The membranes were dried at 37 °C for 2 h. An absorbent membrane was affixed to the nitrocellulose membrane. The resulting test strips were cut into 3.5 mm wide strips using an automatic guillotine ZQ2002 (Shanghai Kinbio Tech, Shanghai, China).

### 3.7. Conjugate Characterization

Conjugate characterization was performed using spectrophotometry, TEM, and DLS as described in Section 3.4. In addition, the oligonucleotide immobilization on nanoparticles was assessed by fluorescence (extinction wavelength 498 nm, emission spectrum from 500 to 600 nm) of the supernatants containing free oligonucleotides using an RF-6000 spectrofluorometer (Shimadzu, Kyoto, Japan). The [Au@Pt]NPs, oligonucleotide, and their conjugate were characterized by FTIR spectrometry. FTIR spectra were recorded in the range of 350–4000 cm^−1^ using an FT/IR-6700 spectrophotometer (JASCO, Tokyo, Japan). All measurements were provided at room temperature. The peroxidase-like activity of the conjugates in solution was determined by a reaction with TMB and hydrogen peroxide as described in Section 3.4.

The functional properties of the conjugates were evaluated using lateral flow test strips (Section 3.6). Conjugates of both types of nanoparticles were normalized by optical density: all [Au@Pt]NP conjugates were adjusted to A_500_ = 0.2. The conjugates were then diluted 10-fold in PBST (final volume 100 μL); for each measurement, two repetitions were made. [Au@Pt]NP-An-ssDNA conjugates were further titrated in PBST in step 3. The test strips were then placed in the resulting conjugate solutions, removed after 3 min, and scanned. Test strips with [Au@Pt]NP conjugates were further washed in 100 μL of PBST (immersed in PBST solution for 5 min), after which 2 μL of the DAB-based substrate solution was added to the binding zone, and the test strips were scanned after 7 min.

### 3.8. Software

Digital images of TEM were analyzed using the Image Tool 3.00 software (University of Texas, Health Science Center, San Antonio, TX, USA). The color intensity of the scanned lateral flow test strips was evaluated using TotalLab TL120 1D v2009 software (Nonlinear Dynamics, Newcastle upon Tyne, UK). Data were processed and visualized using OriginPro 2019b software (OriginLab, Northampton, MA, USA).

## 4. Conclusions

For the first time, a comprehensive study related to the freeze–thaw method for immobilizing non-thiolated DNA oligonucleotides on the surface of [Au@Pt]NPs was performed. All previously used methods were based on the immobilization of thiolated DNA oligonucleotides on the [Au@Pt]NPs’ surface using methods such as salt-aging and low-pH immobilization and freeze–thaw adsorption (a comparative table is presented in the Supporting Information, Table S2). The proposed method of oligonucleotide immobilization relied on a polyA anchor at the 5′-end for attachment to the nanoparticle surface, coupled with a polyT block at the opposite end to stabilize the oligonucleotide on the nanoparticle. The absence of the need for a thiol group provides an immobilization procedure that is significantly cheaper (considering the synthesized oligonucleotides) and time-saving (because of it does not require additional treatment to activate disulfide bonds). We demonstrated that the effectiveness of the resulting conjugates, in terms of stability and functionality, depends on the length and structure of the polyA anchor. The conjugates containing A_5_, A_7_, and 3A_3_-ssDNA showed lower aggregation tendencies and higher signals (both optical and catalytic) in lateral flow assays. These findings contrast with the previously established optimal polyA block structure (A_10_) for oligonucleotide conjugation with [Au]NPs, which was confirmed again in this study. The long (A_10_) or highly branched (3A_5_) polyA blocks were not optimal for functionalizing [Au@Pt]NPs. For [Au@Pt]NPs, the spiky and rough surface likely hinders the binding of longer polyA blocks, whereas shorter polyA sequences (A_3_, A_5_, A_7_-ssDNA) demonstrated stronger and more stable binding to the Pt surface compared to the Au surface.

These results open new avenues for efficient syntheses of [Au@Pt]NP–oligonucleotide conjugates. Such conjugates represent powerful tools at the intersection of nanotechnology and biotechnology, with potential applications across a wide range of fields, from diagnostics to bioanalysis. The obtained results provide a solid methodological foundation for the subsequent syntheses of Pt-containing nanozyme–oligonucleotide conjugates and have broad applications in various bioanalytical systems. The peroxidase-like activity of [Au@Pt]NPs provides greater sensitivity of analyses in comparison with the systems based on colorimetric detection of [Au]NPs or [Au@Pt]s. Therefore, the proposed conjugates can be used for bioanalytical systems such as LFA where recognition of DNA fragments (due to the oligonucleotide part of the conjugate) and high detection sensitivity (due to the peroxidase-like activity of [Au@Pt]NPs in the conjugate) are required.

## Figures and Tables

**Table 1:** Sequences of ssDNA oligonucleotides used in this study.

Name	Sequence 5′-3′
A_3_-ssDNA	AAACCTCCAAGAGTTAGATCATACAGTTTTTTT-FAM *
A_5_-ssDNA	AAAAACCTCCAAGAGTTAGATCATACAGTTTTTTT-FAM
A_7_-ssDNA	AAAAAAACCTCCAAGAGTTAGATCATACAGTTTTTTT-FAM
A_10_-ssDNA	AAAAAAAAAACCTCCAAGAGTTAGATCATACAGTTTTTTT-FAM
3A_3_-ssDNA	(AAA)_3_[TREBLER]CCTCCAAGAGTTAGATCATACAGTTTTTTT-FAM **
3A_5_-ssDNA	(AAAAA)_3_[TREBLER]CCTCCAAGAGTTAGATCATACAGTTTTTTT-FAM

* FAM—fluorescein; ** [TREBLER]—branch point in 5′-terminal direction due to using trebler phosphoramidite for synthesis of oligonucleotides.

## Data Availability

The data presented in this study are available on request from the corresponding author.

## Supplementary Materials

The following supporting information can be downloaded at https://www.mdpi.com/article/10.3390/ijms251810108/s1.
