# Supplementary material for: Freeze-Driven Adsorption of Oligonucleotides with polyA-Anchors on Au@Pt Nanozyme
Source: Int J Mol Sci. 2024 Sep 20;25(18):10108. doi: 10.3390/ijms251810108 (PMC11432674; doi:10.3390/ijms251810108)
Supplement: Supplementary file 1 [file ijms-25-10108-s001.zip › ijms-3195899-supplementary.pdf]

Freeze-Driven Adsorption of Oligonucleotides with polyA-Anchors on Au@Pt Nanozyme

Nikita E. Lapshinov, Svetlana M. Pridvorova, Anatoly V. Zherdev, Boris B. Dzantiev and Irina V. Safenkova *

A.N. Bach Institute of Biochemistry, Research Centre of Biotechnology of the Russian Academy of Sciences, 119071 Moscow, Russia; nikita_lapshinov@mail.ru (N.E.L.); sh-p_s@mail.ru (S.M.P.); zherdev@inbi.ras.ru (A.V.Z.); dzantiev@inbi.ras.ru (B.B.D.)

***** Correspondence: safenkova@inbi.ras.ru; Tel./Fax: +7-495-954-2804

CONTENT

Figure S1. Diameter distributions of [Au]NPs and [Au@Pt]NPs by TEM ……………………………………………2

Figure S2. Characterization of the catalytic activity of [Au@Pt]NPs and [Au@Pt]NP–A10-ssDNA conjugate ……3

Figure S3. Emission spectra obtained for three supernatants after three consecutive centrifugations of conjugates ……4

Figure S4. Characterization of [Au@Pt]NPs, A_{10}-ssDNA oligonucleotide and their conjugate using FTIR technique ……5

Figure S5. TEM micrographs of [Au]NP–A_n-ssDNA conjugates ……………………………………………………6

Figure S6. TEM micrographs of [Au@Pt]NP–A_n-ssDNA conjugates ………………………………………………7

Figure S7. Test strips colored due to the colorimetric properties of [Au]NPs ……………………………………8

Figure S8. Dependencies of binding zone coloration of teat strips on [Au]NP–A_n-ssDNA conjugate dilution ……9

Figure S9. Test strips colored due to the colorimetric properties of nanozyme and the signal enhancement based on catalytic properties of nanozyme ……………………………………………………………10

Table S1. Hydrodynamic characteristics of the conjugates ……………………………………………………………12

Table S2. Methods for immobilization of oligonucleotides on the surface of Pt-containing nanoparticles………13

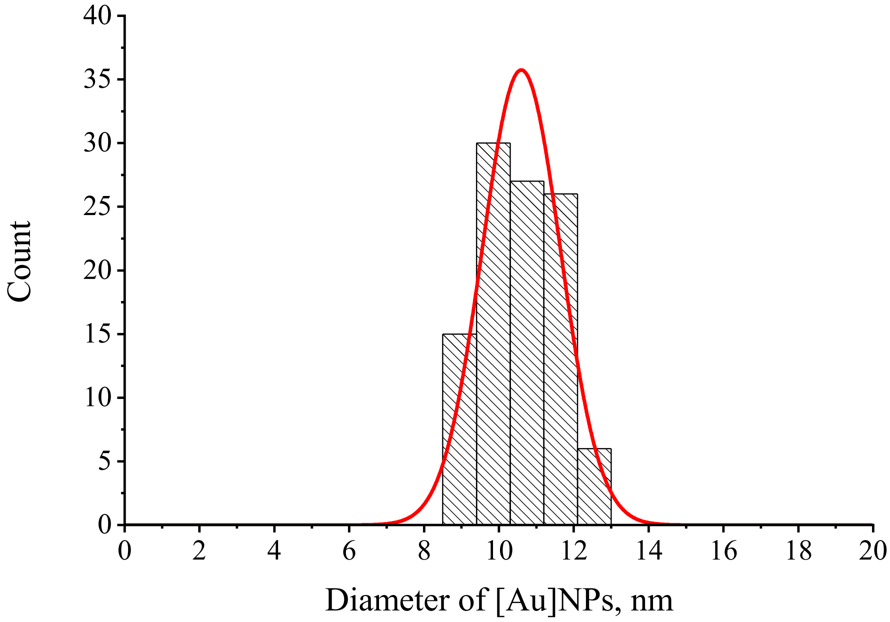

A

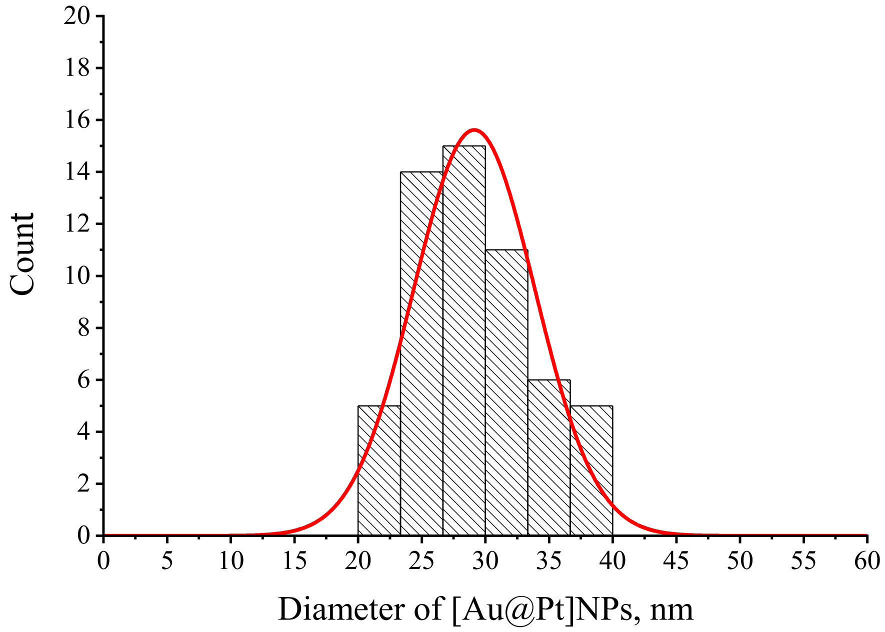

B

Figure S1. Diameter distributions of [Au]NPs (A) and [Au@Pt]NPs (B) by TEM.

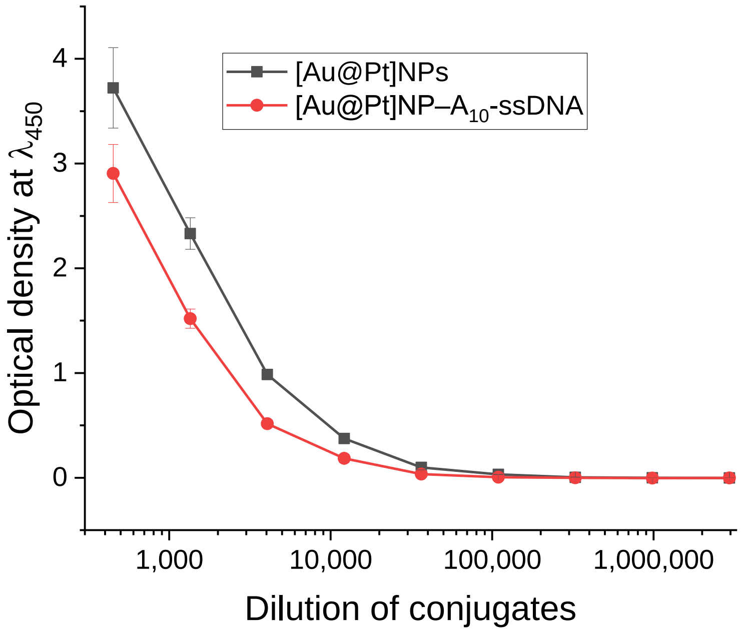

Figure S2. Characterization of the catalytic activity of [Au@Pt]NPs and [Au@Pt]NP–A_{10}-ssDNA conjugate in the reaction with 3,3',5,5'-tetramethylbenzidine and hydrogen peroxide.

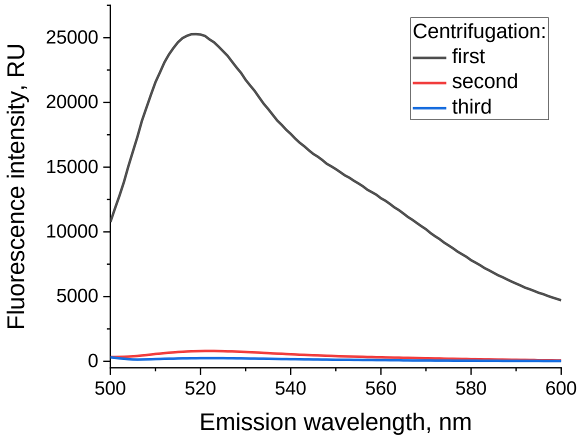

(**A**)

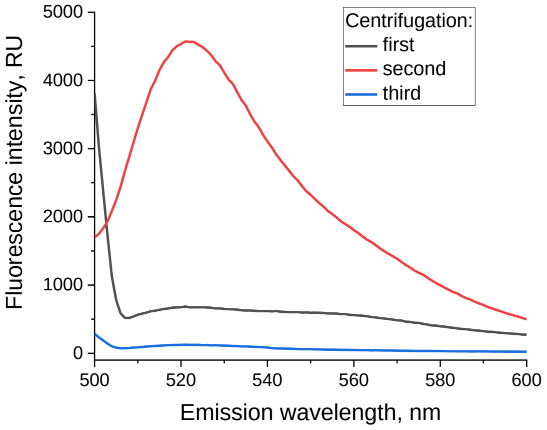

(**B**)

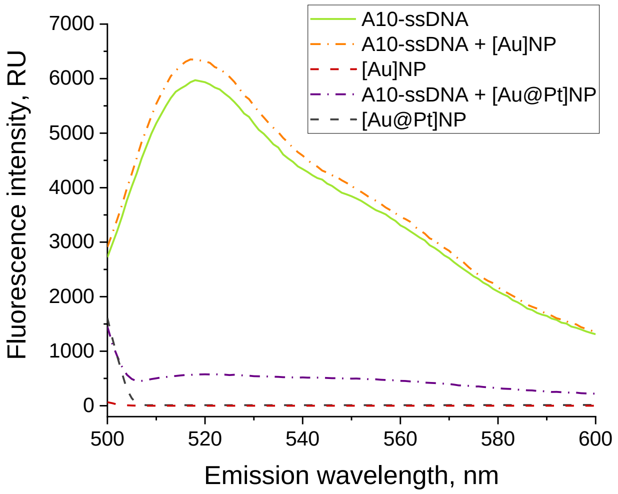

(**C**)

Figure S3. Emission spectra obtained at an extinction wavelength of 498 nm for **(A)** three supernatants after three consecutive centrifugations of [Au]NP–A_{10}-ssDNA conjugates, **(B)** three supernatants after three consecutive centrifugations of [Au@Pt]NP–A_{10}-ssDNA conjugates, (**C**) for individual components (A_{10}-ssDNA, [Au]NP, [Au@Pt]NP) and their mixtures (A_{10}-ssDNA + [Au]NP, A_{10}-ssDNA + [Au@Pt]NP).

[Au@Pt]NPs absorb strongly in the entire visible light range, so even a small amount of them in the supernatant (e.g., a fraction of the smallest particles that do not settle during centrifugation) results in strong absorption at the measured fluorescein extinction wavelength (498 nm) (**Figure S3-B, C**). After the second centrifugation of the [Au@Pt]NP–A_{10}-ssDNA conjugate, there are no [Au@Pt]NPs in the supernatant, so recording the remaining free oligonucleotide with a fluorescein label becomes possible (**Figure S3-B**). [Au]NPs do not have such an effect on fluorescence in the fluorescein range (**Figure S3-C**).

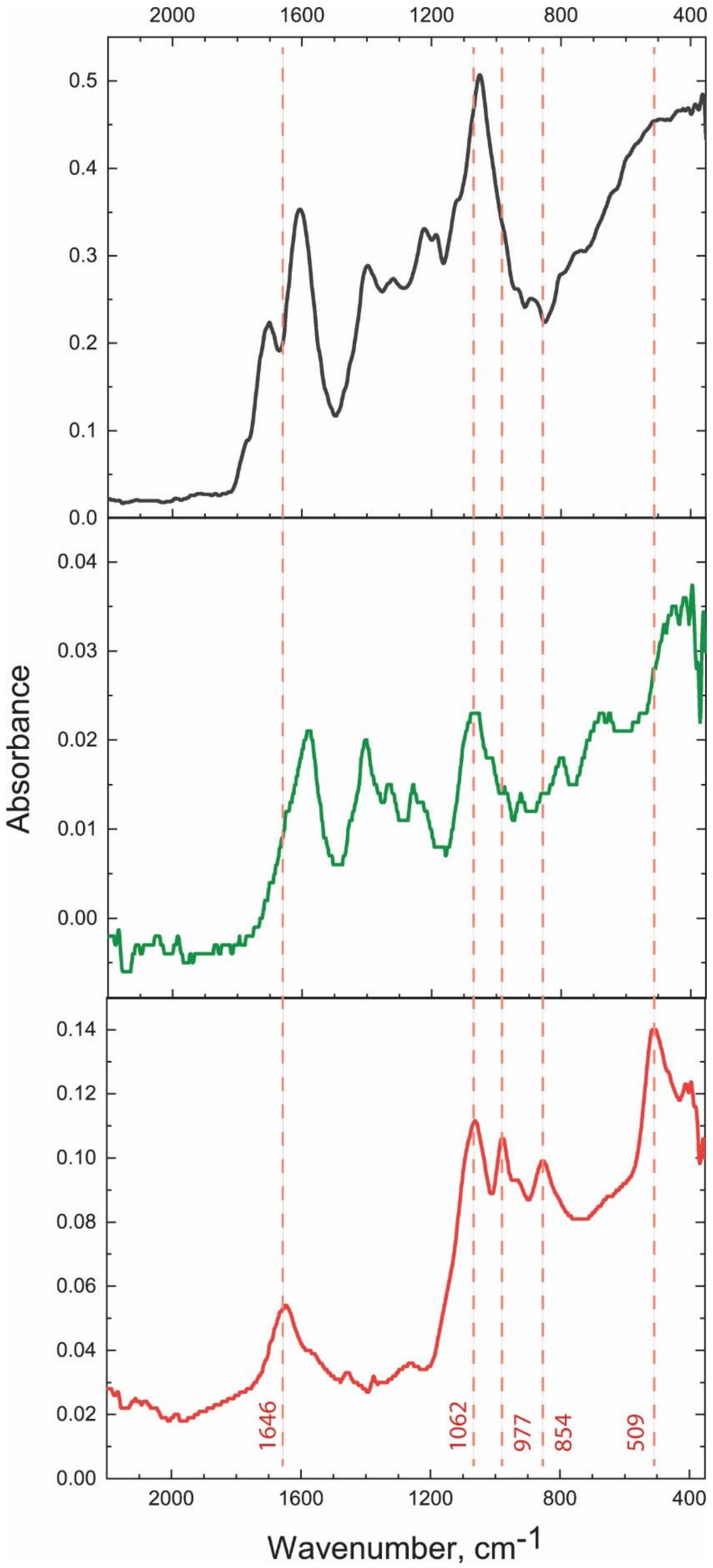

Figure S4. Characterization of [Au@Pt]NPs (black line), A_{10}-ssDNA oligonucleotide (green line) and their conjugate (red line) using FTIR technique.

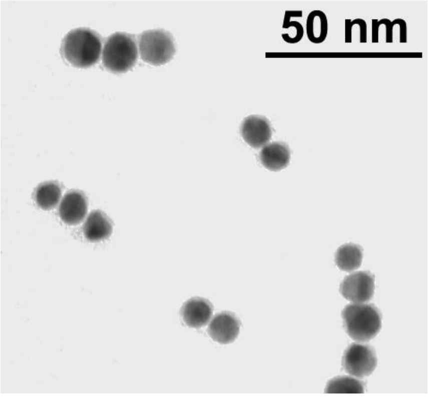

(A)

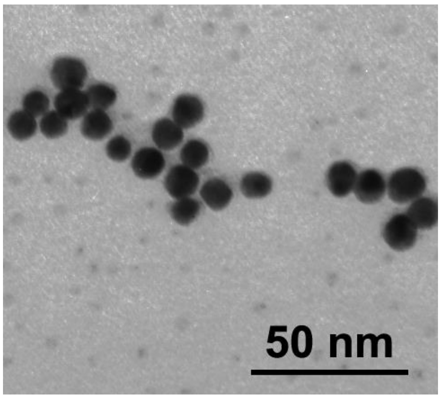

(B)

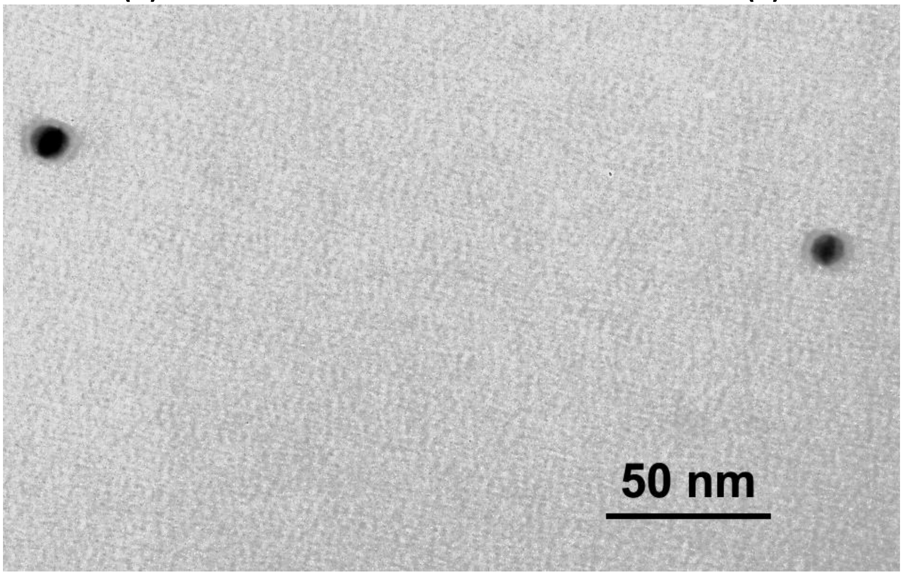

(C)

Figure S5. TEM micrographs of [Au]NP–A_5-ssDNA (**A**), [Au]NP–A_7-ssDNA (**B**), [Au]NP–3A_5-ssDNA (**C**).

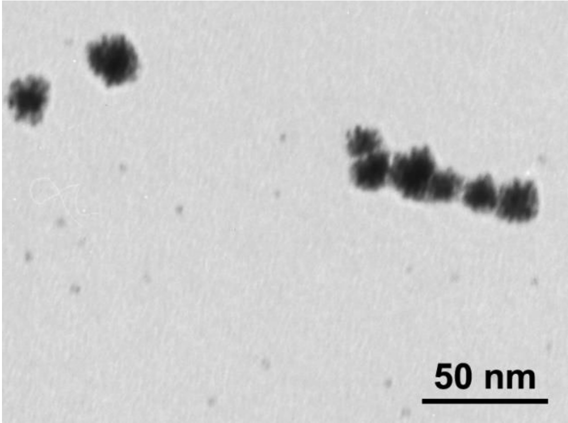

(A)

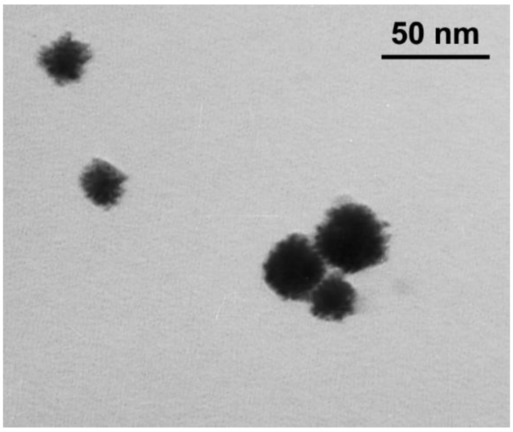

(B)

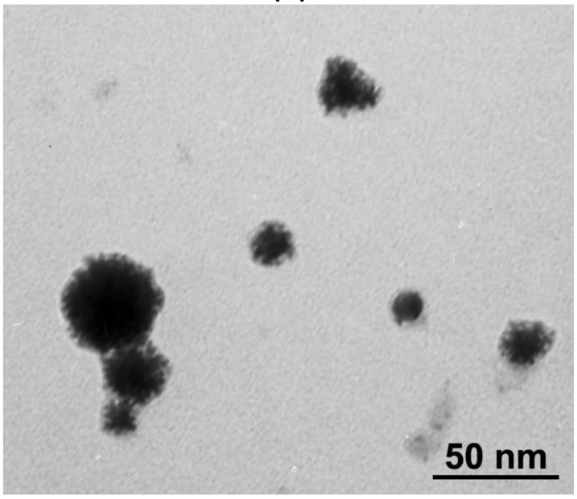

(C)

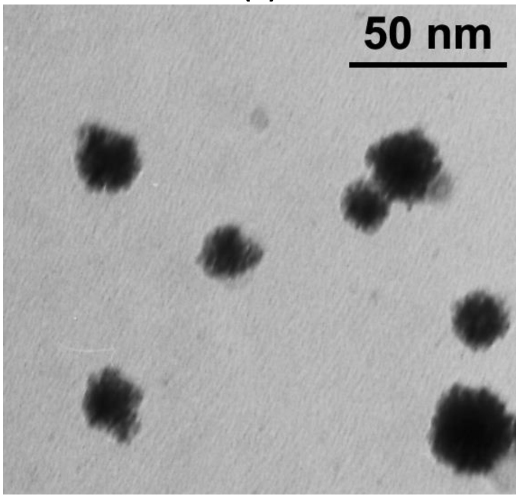

(D)

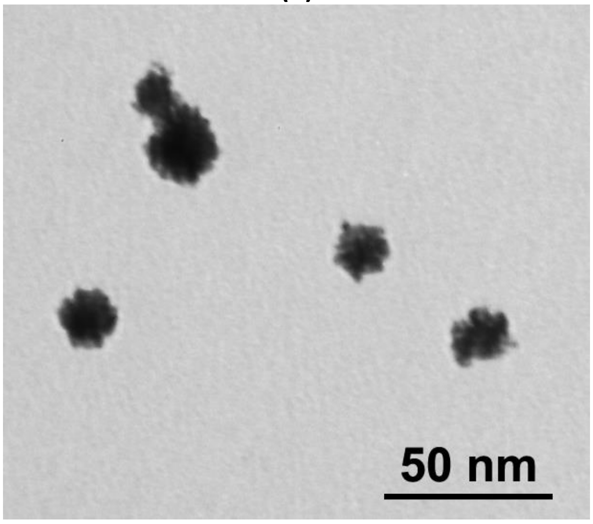

(E)

Figure S6. TEM micrographs of [Au@Pt]NP–A_3-ssDNA (**A**), [Au@Pt]NP–A_5-ssDNA (**B**), [Au@Pt]NP–A_{10}-ssDNA (**C**), [Au@Pt]NP–3A_3-ssDNA (**D**), [Au@Pt]NP–3A_5-ssDNA (**E**).

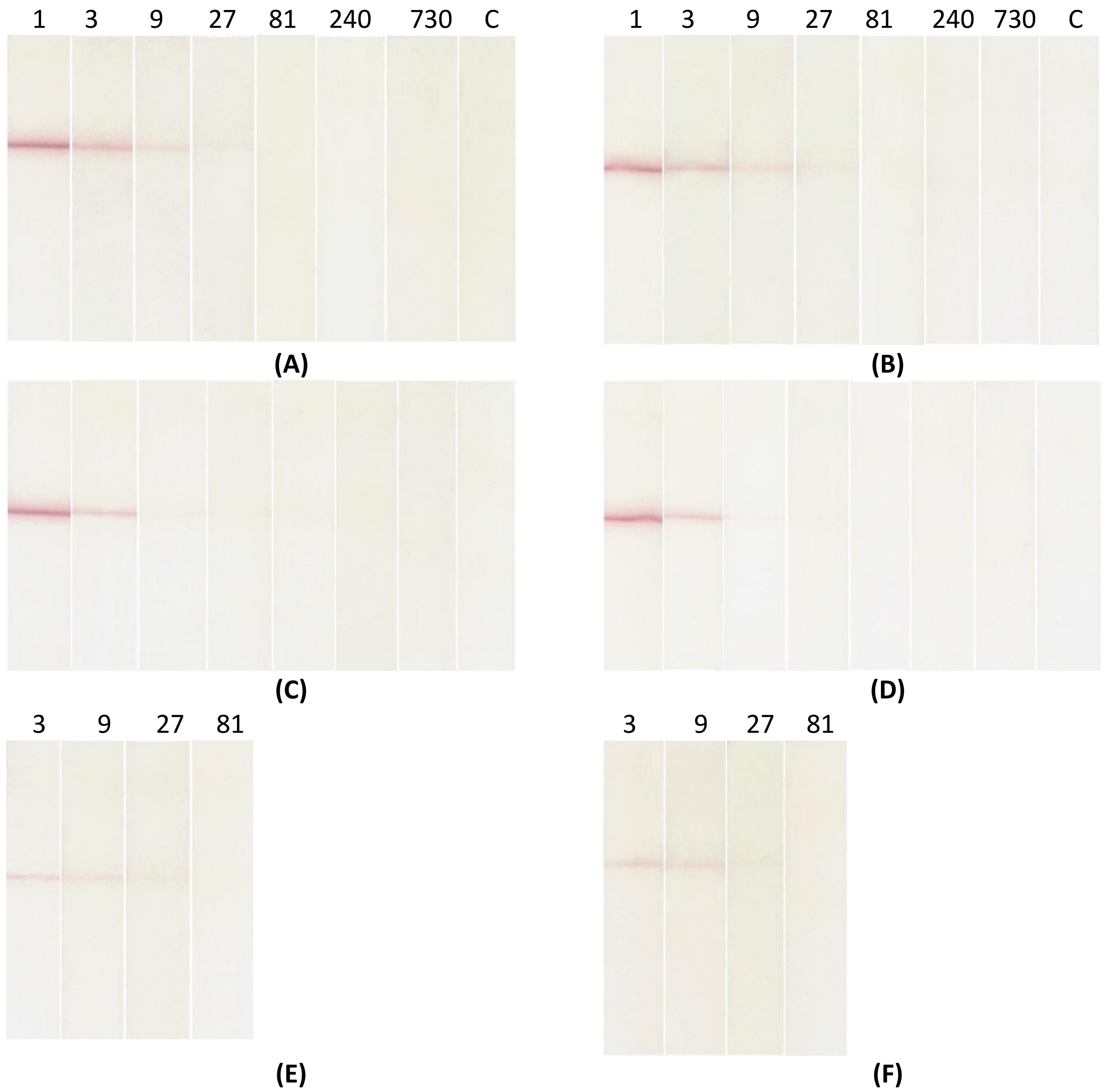

Figure S7. Test strips colored due to the colorimetric properties of nanozyme for [Au@Pt]NP–A_7-ssDNA (**A**), [Au@Pt]NP–A_{10}-ssDNA (**B**), [Au@Pt]NP–$3A_3$-ssDNA (**C**), [Au@Pt]NP–$3A_5$-ssDNA (**D**), [Au]NP–A_3-ssDNA (**E**), [Au@Pt]NP–A_5-ssDNA (**F**). The numbers indicate the dilution factor, C shows the control experiment without conjugate.

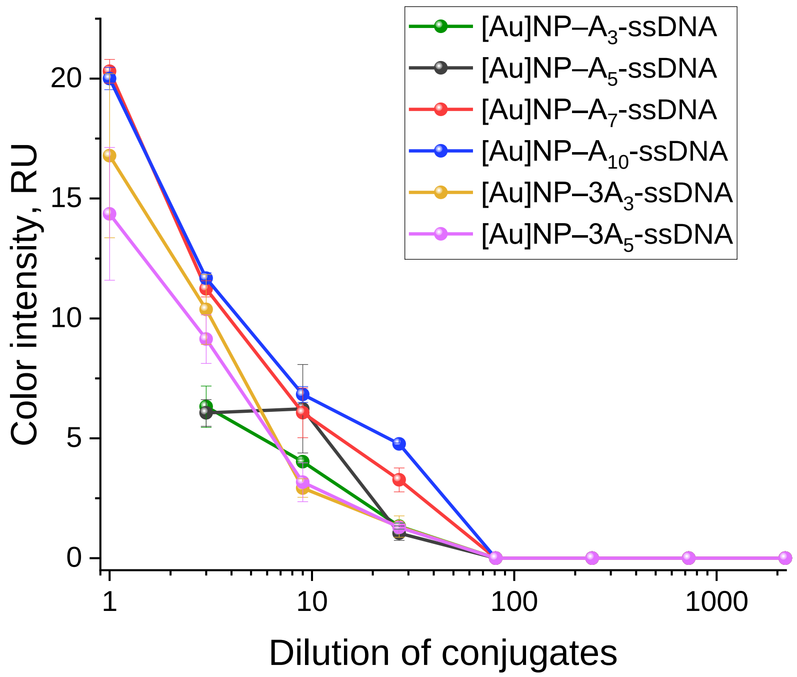

Figure S8. Dependencies of binding zone coloration of teat strips on [Au]NP–An-ssDNA conjugate dilution. For each dilution, two replicates were made, the figure shows the mean values and standard deviations as error bars.

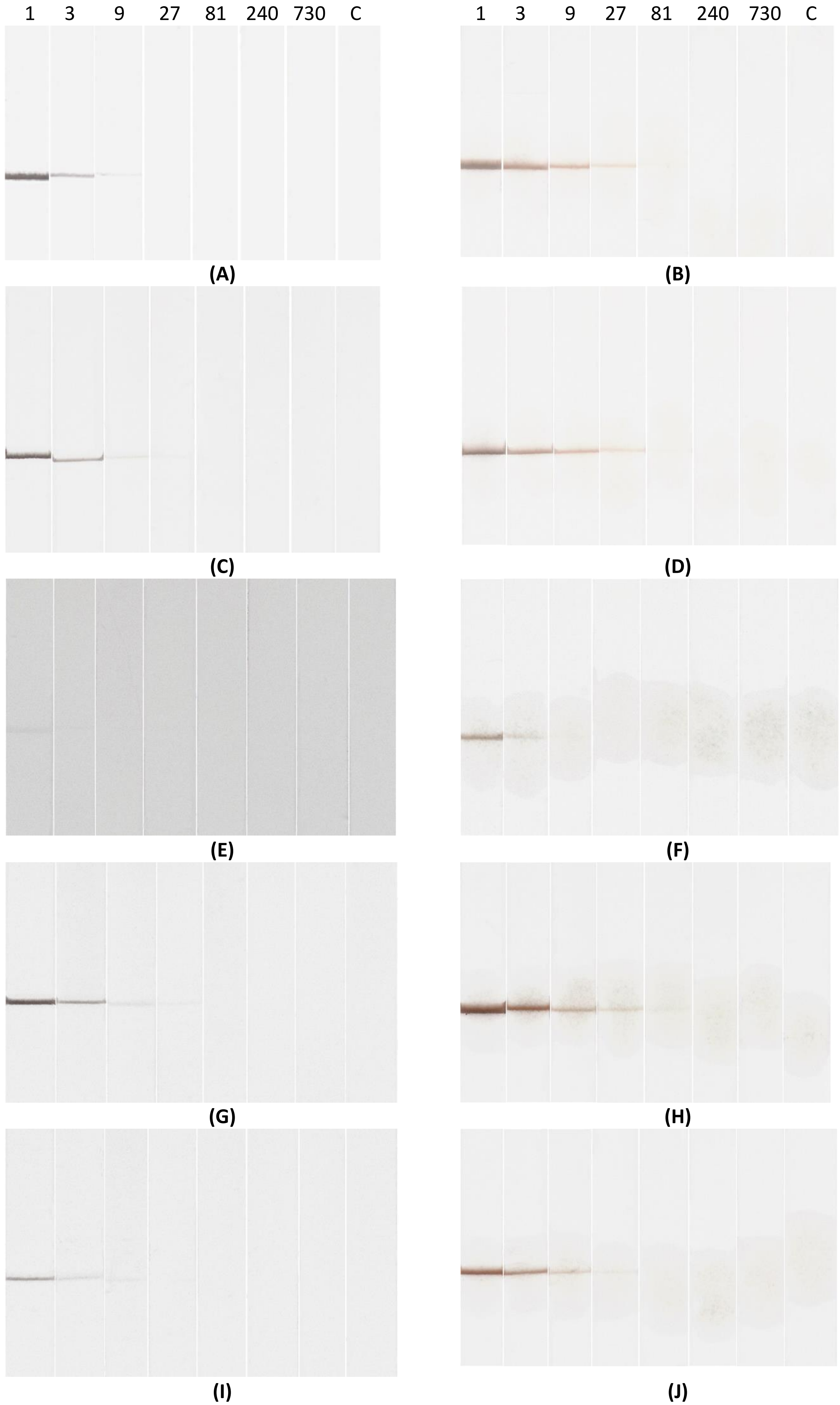

Figure S9. Test strips colored due to the colorimetric properties of nanozyme for [Au@Pt]NP–A_3-ssDNA (**A**), [Au@Pt]NP–A_5-ssDNA (**C**), [Au@Pt]NP–A_{10}-ssDNA (**E**), [Au@Pt]NP–3A_3-ssDNA (**G**), [Au@Pt]NP–3A_5-

ssDNA (**I**). Test strips colored due to colored due to the signal enhancement based on catalytic properties of nanozyme for [Au@Pt]NP–A_3-ssDNA (**B**), [Au@Pt]NP–A_5-ssDNA (**D**), [Au@Pt]NP–A_{10}-ssDNA (**F**), [Au@Pt]NP–3A_3-ssDNA (**H**), [Au@Pt]NP–3A_5-ssDNA (**J**). The numbers indicate the dilution factor, C shows the control experiment without conjugate.

Table S1. Hydrodynamic characteristics of the conjugates

Conjugate		Average hydrodynamic diameter, nm	Standard deviation, nm	Polydispersity index, %
[Au]NPs	A_5-ssDNA	21.1	6.9	0.330
	A_7-ssDNA	23.9	7.6	0.43
	A_{10}-ssDNA	19.2	5.7	0.232
	$3A_5$-ssDNA	21.0	5.9	0.300
[Au@Pt]NPs	A_3-ssDNA	37.5	10	0.313
	A_5-ssDNA	44.5	12.4	0.115
	A_7-ssDNA	39.2	11.7	0.154
	A_{10}-ssDNA	45.4	12.3	0.154
	$3A_3$-ssDNA	43.4	11.9	0.202
	$3A_5$- ssDNA	41.6	14.1	0.271

Table S2. Methods for immobilization of oligonucleotides on the surface of Pt-containing nanoparticles

No	Pt-containing nanoparticle	Oligonucleotide	Immobilization method	Functional group in oligonucleotide for immobilization	Reference
1	spherical [Au@Pt]NPs	Cy3-DNAzyme-HS	freeze-thaw method	-SH	[1]
2	spherical [Au@Pt]NPs	HS-ssDNA-Cy3	freeze-thaw method	-SH	[2]
3	spherical [Au@Pt]NPs	5′-SH-$(CH_2)_6$-AAAAAAAAAACGC AGT AAT GAC GTC GAC ATC ATA	salt-aging immobilization	-SH	[3]
4	spherical [Au@Pt]NPs	disulfide-labeled ssDNA	salt-aging immobilization	-SH	[4]
5	urchin-like [Au@Pt]NPs	series of non-thiolated ssDNA	freeze-thaw method	5′-polyadenine anchor (A_n, with n = 3, 5, 7, 10; triple-branched A_3, and triple-branched A_5)	this study

[1] Lei, Y.; He, X.; Zeng, Y.; Wang, X.; Yang, L.; Liu, X.; Qing, Z., Pt–S bond stabilized DNAzyme nanosensor with thiol-resistance enabling high-fidelity biosensing. Talanta 2024, 276, 126187.
[2] Qing, Z.; Luo, G.; Xing, S.; Zou, Z.; Lei, Y.; Liu, J.; Yang, R., Pt–S Bond-Mediated Nanoflares for High-Fidelity Intracellular Applications by Avoiding Thiol Cleavage. Angewandte Chemie International Edition 2020, 59, (33), 14044-14048.
[3] Chen, G.; Jin, M.; Yan, M.; Cui, X.; Wang, Y.; Zheng, W.; Qin, G.; Zhang, Y.; Li, M.; Liao, Y.; Zhang, X.; Yan, F.; Abd El-Aty, A. M.; Hacimuftuoglu, A.; Wang, J., Colorimetric bio-barcode immunoassay for parathion based on amplification by using platinum nanoparticles acting as a nanozyme. Mikrochimica acta 2019, 186, (6), 339.
[4] Shao, N.; Han, X.; Song, Y.; Zhang, P.; Qin, L., CRISPR-Cas12a Coupled with Platinum Nanoreporter for Visual Quantification of SNVs on a Volumetric Bar-Chart Chip. Anal Chem 2019, 91, (19), 12384-12391.
